# Oral biopsies in a Portuguese population: A 20-year clinicopathological study in a university clinic

**DOI:** 10.4317/jced.59688

**Published:** 2022-12-01

**Authors:** Cláudia G. de Almeida, Filipe Freitas, Helena Francisco, João A. Marques, João Caramês

**Affiliations:** 1 DDS. Faculdade de Medicina Dentária, Universidade de Lisboa, Lisbon, Portugal; 2 DS, PhD. Faculdade de Medicina Dentária, Universidade de Lisboa, Lisbon, Portugal

## Abstract

**Background:**

Performing a biopsy is very important in oral medicine and the anatomopathological examination is fundamental to obtain or to confirm the diagnosis in oral and maxillofacial pathology. The purpose of this study is to analyse the frequency and characteristic patterns of biopsied oromaxillofacial lesions in a Portuguese population.

**Material and Methods:**

A descriptive statistical analysis of the data from the anatomopathological reports of the biopsies performed between 1999 and 2019 at the university clinic of the Faculty of Dental Medicine of the University of Lisbon was performed, regarding the patient’s gender and age, type of biopsy, location of lesions, clinical and histological diagnosis, and the results were obtained. Association relationships were studied using the chi-square test and the Kruskal-Wallis test to correlate variables. *P*<0.05 was considered statistically significant.

**Results:**

From a total sample of 1448 patients, 826 (57.1%) were female, 610 (42.1%) were male, and 12 (0.8%) had no gender information, with a mean age of 50.14 years (standard deviation ± 17.61). The preferred location was the buccal mucosa, vestibule fundus and alveolar mucosa (20.7%). Benign lesions (BL) were the most common, in 82,8% of the cases, followed by oral potentially malignant disorders (OPMD) in 15,5%, and finally, malignant lesions (ML) in 1.7%. Focal fibrous hyperplasia was the most frequent diagnosis in the total sample (25.6%). In the young group, the most common entity was mucocele (34.0%), with a predominance of the lower lip (32.9%). In OPMD, leukoplakia was the most frequently diagnosed (48,7%). The most common ML was squamous cell carcinoma (92.0%), appearing mainly in the tongue (34.8%). A statistically significant relation between ML and older age was found.

**Conclusions:**

This study included biopsies analysed over a period of 20 years, being BL the main pathology to affect the oral cavity. Although less frequent, OPMD and ML should not be neglected and must be correctly diagnosed and treated.

** Key words:**Oral biopsies, Oral and maxillofacial pathology, Oral medicine, Clinicopathological analysis, Epidemiological study, University clinic.

## Introduction

The dentist is the health professional responsible for the study, prevention, diagnosis and treatment of anomalies and diseases of the teeth, mouth, jaws and adjacent structures, integrating the patient’s multidisciplinary approach. He has also an increasingly proactive role in the daily lives of the population, being often the first to detect pathologies, such as oral cancer, that require specialized attention and treatment. Oral medicine is concerned with the diagnosis and medical management of specific diseases of the orofacial tissues, as well as with the treatment of oral manifestations of systemic conditions (1,2).

The lesions of the oral cavity cover a wide spectrum regarding their nature and characteristics, which can difficult their diagnosis, sometimes (3). Therefore, performing a biopsy is an important tool for the dentist. It is a surgical procedure that aims to obtain tissue from a living individual for histopathological analysis. The pathological examination helps to define the diagnosis, facilitates the determination of the prognosis of malignant lesions, contributes to the institution of treatment or evaluation of its effectiveness, and constitutes a document with legal medical value (4).

Epidemiological studies provide an overview of the lesions that are most frequently found in the doctor’s office, which is why they are important methods of investigation.

This epidemiological study aimed to evaluate the oral pathologies most frequently subjected to biopsy in a Portuguese university clinic, therefore being benign or malignant. It was also intended to analyse the demographic and clinical characteristics of pathological entities with indication for histological diagnosis.

## Material and Methods

The present study consists of an observational epidemiological study of the last 20 years. Data related to the patient’s gender and age, type of biopsy performed, anatomical location of lesions, clinical and histological diagnosis were collected from the anatomopathological reports of the biopsies performed between 1990 and 2019 at the Faculty of Dental Medicine of the University of Lisbon (FMDUL). Then the information was transferred into a Microsoft Excel database. The lesions that were not located in the oromaxillofacial region and without a well-defined histological diagnosis were excluded.

The pathologies were classified according to the International Classification of Diseases, Dentistry and Stomatology (ICD-11), and by the fourth edition of the World Health Organization (WHO) Classification of Head and Neck Tumours (5). We divided the lesions into three groups for analysis: benign lesions (BL); oral potentially malignant disorders (OPMD) according to the WHO 2020 classification; and malignant lesions (ML) (6).

Ages were distributed into age groups at 10-year intervals. For study, they were also further divided into three age groups: young group (0-17 years); adult group (18-64 years) and elderly group (≥65 years). The type of biopsy was considered excisional, incisional, and unspecified. The anatomical locations of the lesions were grouped into categories for further analysis. Finally, the correlation between the respective clinical and histological diagnoses was also analysed.

Statistical analysis was performed using SPSS 27.0 Data Editor (SPSS Inc., Chicago, USA). The results describe the absolute and relative frequencies, as well as study the possible associations between variables. Association relationships were studied using the chi-square test to correlate qualitative variables and the Kruskal-Wallis test to correlate age and some qualitative variables. *P*<0.05 was considered statistically significant.

## Results

From an initial total of 1623 biopsies, 175 were excluded for not belonging to the target region or for not having a histological diagnostic record, resulting in a sample of 1448 cases.

From a final sample of 1448 cases analysed, 12 (0.8%) had no gender information, 260 (18%) had no age information, 2 (0.1%) had no record of the location of the biopsied lesion and 214 (18.6%) had no record of provisional clinical diagnosis hypotheses.

Of the sample, 57.1% were female patients and 42.1% male patients, corresponding to a total of 826 and 610 biopsies, respectively.

The patient’s age ranged between 4 and 91 years, with a mean age ± standard deviation of 50.14 ± 17.61 years. The most common age group was from 50 to 59 years old, representing 23,5%. In terms of valid percentage, it was observed that 4.5% of the sample belonged to the young group, 72.3% to the adult group and 23.2% to the elderly group.

Of 1448 biopsied lesions, 1199 (82,8%) corresponded to benign lesions, 224 (15,5%) to oral potentially malignant disorders and 25 (1,7%) to malignant lesions. Despite the preference for the female gender in all types of pathologies, there was no statistically significant relationship between nature of lesion and gender (*P* > 0.5). It was found that the age in ML is significantly higher than the age of the other two types of lesions (*P*=0.000). It was observed that ML manifested preferentially in the tongue, while the other types of lesions occurred mainly in buccal mucosa, as seen in Table 1.

**Table 1:**
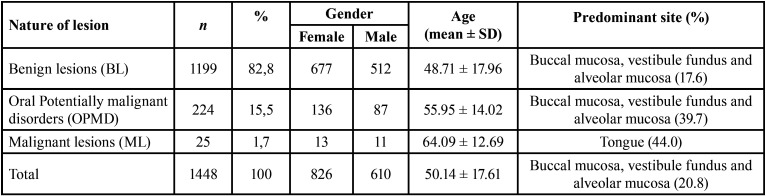
Absolute and relative frequency of each type of lesion and respective distribution of gender, age, and predominant anatomical location.

There were 85 different diagnoses, and the most frequent was focal fibrous hyperplasia, also known as traumatic fibroma (25.62%). This pathology revealed an average age of 52.70 years, occurring mainly in the buccal mucosa (28.6%). The radicular cyst was the most diagnosed hard tissue lesion, mostly located in the anterior region of the maxilla. Leukoplakia and oral lichen planus were the most frequent oral potentially malignant disorders, occupying the third and fourth place respectively. Both with preferential manifestation in the buccal and alveolar mucosa, and at the vestibule fundus. It should also be noted that there was a higher preponderance of the female gender with oral lichen planus. In eleventh place, there is the squamous cell carcinoma, being the most diagnosed malignant lesion, without gender differences in this study, with an average age of 63 years, and with predominant manifestation in the tongue. The Table 2 provides all the information, regarding frequency, gender, age and predominant site, about the top 20 diagnoses.

**Table 2:**
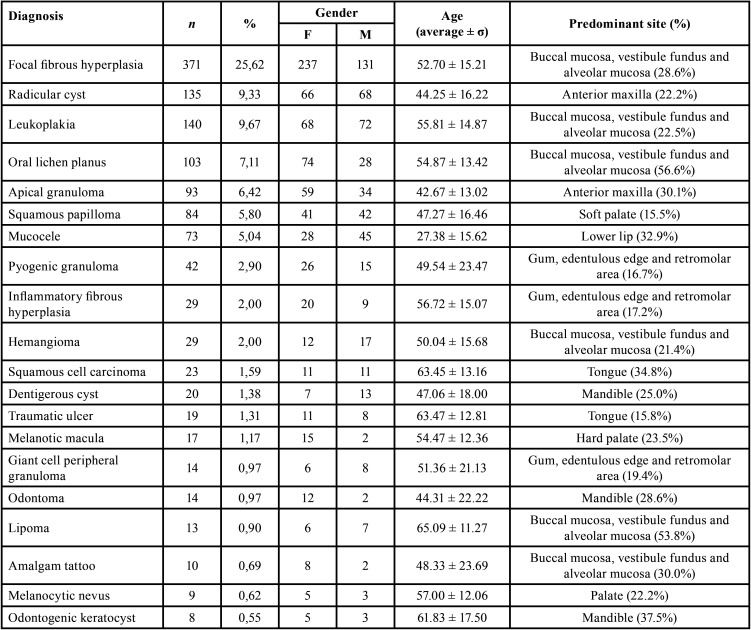
Absolute and relative frequency of the 20 most common diagnoses and respective distribution of gender, age, and predominant anatomical location.

**Figure 1:**
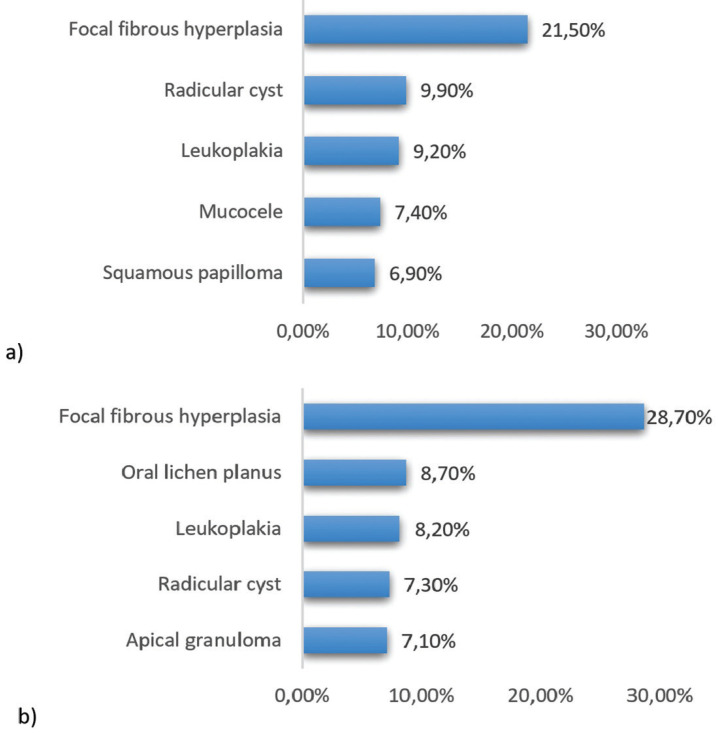
The 5 most common histological diagnoses according to gender: a) male and b) female.

Focal fibrous hyperplasia was the most diagnosed entity in both genders, 21.5% in males and 28.7% in females. Although the pathologies diagnosed in each gender are very similar, in females we observe a greater number of OPMD (Fig. 1).

**Figure 2:**
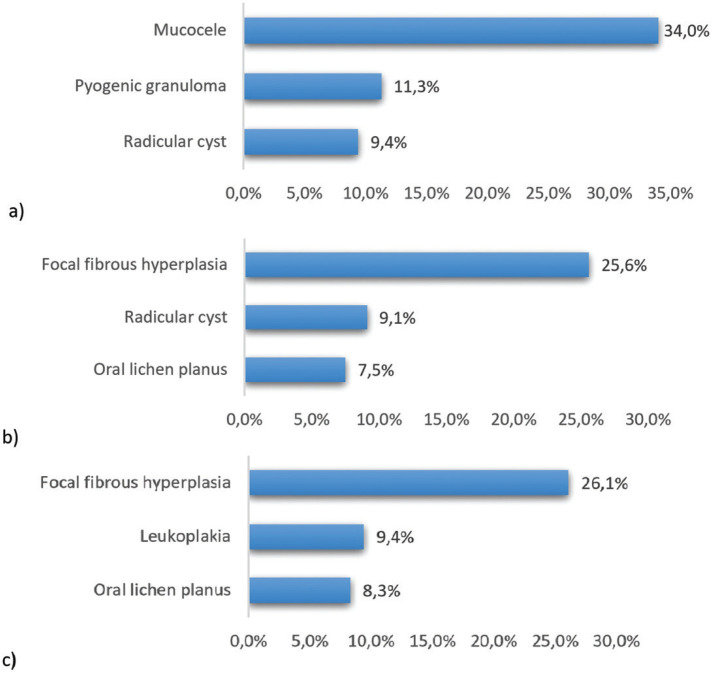
The 3 most common histological diagnoses according to the age group: a) young patients, b) adult patients, and c) elderly patients.

When we organized patients into age groups, we found that in the young group 34.0% of the lesions corresponded to mucoceles. Focal fibrous hyperplasia was the most frequent lesion both in the adult group, occupying 25.6% of the biopsied lesions, as well as in the elderly group with a percentage of 26.1% (Fig. 2).

The most frequent location was the buccal mucosa, vestibule fundus, and alveolar mucosa with 300 associated lesions (20.7%), with the tongue being the second most frequent location with 210 lesions (14.5%). More information about the frequency according to anatomical site is written on the Table 3.

**Table 3:**
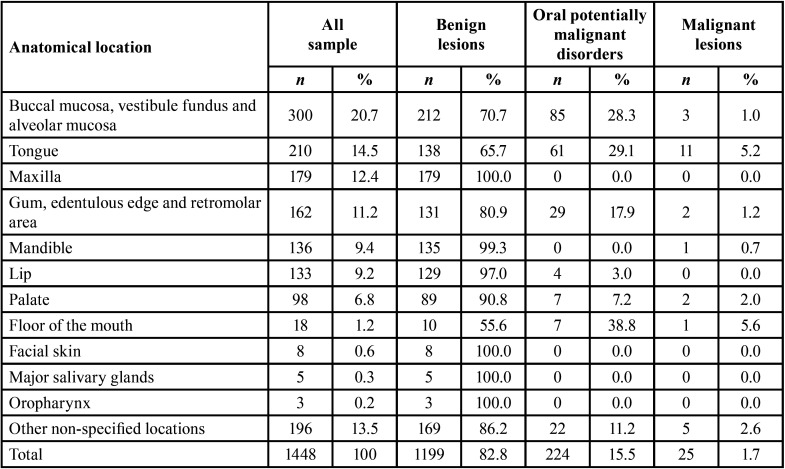
Frequency of lesions according to anatomical location.

From a total of 1448 biopsies performed, 68.09% were excisional biopsies, 16.44% incisional biopsies and 15.47% did not have this specified information.

**Figure 3:**
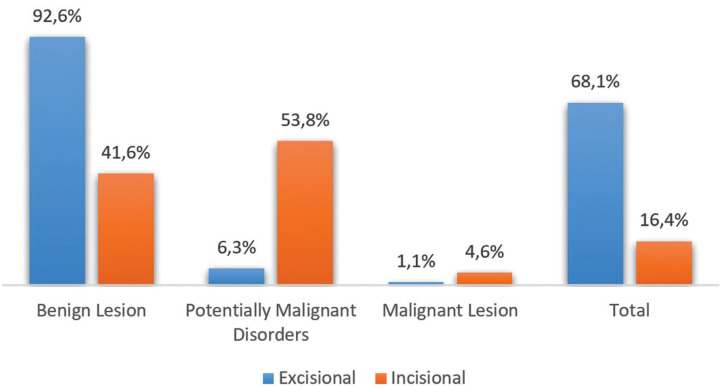
Type of biopsy according to the nature of lesion.

Another fact that was found was that when an incisional biopsy was performed, the percentage of oral potentially malignant disorders was higher than the percentage of benign lesions, contrary to what happens in excisional biopsies. It should also be noted that, in malignant lesions, the preferred type of biopsy was incisional, unless the lesion is smaller than 1cm (Fig. 3).

In most of the cases studied, there is a correlation between the clinical diagnosis and the histological diagnosis, with a total of 902 concordant cases, making up 62.3% of the total study sample. On the other hand, there was no correlation in 37.7% (n=546) of the cases.

The group of OPMD revealed a higher percentage of clinical-pathological agreement, 80.4% (n=177), with only 19.6% of non-concordant cases (n=47), contrary to what is seen in the group of malignant lesions, where there is the lowest percentage of agreement between the diagnoses, 44.0% (n=11), and 56.0% (n=14) non-concordant.

**Figure 4:**
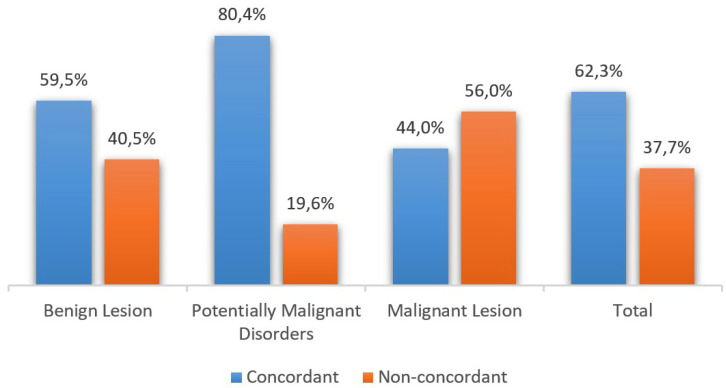
Clinical-pathological concordance in different types of lesions.

On the other hand, about benign lesions, in 59.5% of the cases there was confirmation of the clinical diagnosis by histological study (n=714), and in 40.5% of the biopsies (n=485) this agreement was not verified (Fig. 4).

## Discussion

Lesions in the oral and maxillofacial region cover a wide range of alterations that may be imperceptible to the patient or have serious implications for their quality of life (7,8). It is important for the dentist to be aware of the most common aspects for his daily clinical practice, being able to diagnose simple diseases and conditions as well as to detect more complex situations. Therefore, it is essential to have epidemiological studies that describe both the frequency of oromaxillofacial lesions and their predominant characteristics (3,7).

This study involved 1448 cases, describing biopsies from 1999 to 2019, performed at Faculty of Dental Medicine of the University of Lisbon. Although the sample turns out to be smaller than that used by Monteiro *et al*. (3), where 3212 oral biopsies performed over a 16-year period were analysed at the Oporto Hospitalar Center, the present study is the largest carried out in a university environment, to date.

The analysis showed a slightly higher frequency of oromaxillofacial lesions in females compared to males, which agrees with other studies (3,9,10). This could be explained on the one hand by the larger female population in Portugal, such as demonstrated in the results of the 2011 Census, and on the other by the fact that there is greater care and concern with oral health on the part of women (11). This frequency was found for all types of injury, in contrast to what is found in other studies, regarding oral potentially malignant disorders and malignant lesions, in which the male gender was preponderant (3,12). It should also be noted that, although no significant relationship has been demonstrated between gender and the type of lesion, the total number of injuries in the OPMD and ML groups was quite small, so further studies are needed on this possible association.

In the present study, the age ranged between 4 and 91 years, demonstrating that lesions in the oral cavity can appear at any time in life. However, the data showed that the age group with the highest frequency of pathologies was from 50 to 59 years old. Regarding OPMD and ML, there was a greater preponderance at older ages, noting that in the young group only BL were seen as the most common, while in the elderly group ML are already part of the most frequent pathologies. These data are consistent with the literature, where advanced age is an important non-modifiable risk factor for oncological diseases (3,13-18).

In the young group, from 0 to 17 years of age, the mucocele stood out, being more common in males, with preferential location in the lower lip, which is consistent with other data (18,22). This frequency may it will be explained by the fact that earlier ages are more associated with possible trauma that could be the origin of this entity.

Benign lesions were the most common type of lesion in the population studied, and focal fibrous hyperplasia represented 25.6% of the total. This change was more commonly found in female patients in their fifth decade of life. As this is a reactive hyperplasia of fibrous connective tissue, its preferential occurrence in non-keratinized areas, namely the buccal mucosa, can be framed with the fact that these areas are related to factors of trauma, such as occlusal plane and prosthesis limits, which agrees with data found in other epidemiological studies (3,17,19,20).

Regarding OPMD, leukoplakia was more frequent on male patients aged between 50 and 59 years, manifesting itself with special preference in the tongue and in the buccal mucosa, similarly to what is reported in other studies (3,9,10). On the other hand, oral lichen planus was concentrated in female patients aged between aged 50 and 59 years, manifesting in more than half in non-keratinized areas, which is in agreement with the literature (13,19,21).

Squamous cell carcinoma was the most frequent type of cancer in its population group, appearing at an average age of 63 years, manifesting itself preferentially in the tongue, similarly to what has been reported in several studies (3,9,10,12,15,16). However, in the current study, with regard to gender, this disease occurred equally in females and males, in contrast to what is reported in the literature (3,9,10,15,16,23,24). This divergence may be due to the fact that the sample related to cancer diseases is very small, and therefore it is a random sample.

Regarding the type of biopsy, the data showed that an excisional biopsy is predominantly performed, which agrees with what was demonstrated by Sixto-Requeijo and Diniz-Freitas (9). Considering that the size and nature of the lesion, among other factors, influence the taking decision on the type of biopsy to be performed (4), and a higher frequency of benign lesions was verified, which were more associated with small dimensions, this predominance of excisional biopsies is justified. In our study 11 patients (1.1%) with malignant lesions undergo excisional biopsy because the lesions were small or clinically benign.

As for the correlation between clinical diagnosis and histological diagnosis, it was verified in 62.3% of the cases studied, noting that in OPMD there was a higher percentage of confirmation of the diagnosis, contrary to what happens in ML. It should be noted that some of the data were lost, others would be incomplete, so in these situations a ‘Non-Concordance’ was applied, which could bias the results in this regard.

It should be noted that the present study is limited by a relatively small sample, and despite being a public establishment, it refers only to the population attending the University Clinic of FMDUL, mostly residing in the metropolitan area of Lisbon. Additionally, it lacks the registration of essential information for a correct characterization of diseases of the oral cavity, so the extrapolation of its results must be done within its restrictions.

Considering the limitations raised above, there is a need for a study with a broader sample at national level. In this way, it would be possible to characterize the most frequent injuries and their respective patterns in the Portuguese population, to compare with international studies.

## Conclusions

This study included a sample of 1448 biopsies over 20 years, through which it was found that benign pathologies are the ones that most affect the oral cavity, with focal fibrous hyperplasia being the most frequent diagnosis in the study population. This pathology predominantly manifests itself in areas exposed to trauma. Leukoplakia was the prominent oral potentially malignant disorder, manifesting itself in the tongue and other non-keratinized areas. Squamous cell carcinoma was the most frequent malignant lesion, with a higher frequency in the tongue. Both OPMD and ML are associated with older ages. It was found that, in most cases, there is a concordance between the clinical and histological diagnosis.

The present study reinforces that oral medicine is, in fact, very vast, and that for a good clinical practice it is necessary to acquire and deepen some knowledge from pre-graduate training. Thus, this comes with the expectation of promoting the search for knowledge in this area and helping all students and dentists in their daily clinical practice, especially in carrying out differential diagnosis, through the characterization of the most frequent pathologies.
